# Analysis of Nitrogen-Doping Effect on Sub-Gap Density of States in a-IGZO TFTs by TCAD Simulation

**DOI:** 10.3390/mi13040617

**Published:** 2022-04-14

**Authors:** Zheng Zhu, Wei Cao, Xiaoming Huang, Zheng Shi, Dong Zhou, Weizong Xu

**Affiliations:** 1 College of Integrated Circuit Science and Engineering, Nanjing University of Posts and Telecommunications, Nanjing 210023, China; 1219023314@njupt.edu.cn (Z.Z.); 1220024107@njupt.edu.cn (W.C.); 2 School of Communications and Information Engineering, Nanjing University of Posts and Telecommunications, Nanjing 210023, China; shizheng@njupt.edu.cn; 3 National Laboratory of Solid State Microstructures, Nanjing University, Nanjing 210093, China; dongzhou@nju.edu.cn (D.Z.); njuphyxwz@126.com (W.X.)

**Keywords:** a-IGZO TFTs, sub-gap states, nitrogen-doping, numerical simulation, stability

## Abstract

In this work, the impact of nitrogen doping (N-doping) on the distribution of sub-gap states in amorphous InGaZnO (a-IGZO) thin-film transistors (TFTs) is qualitatively analyzed by technology computer-aided design (TCAD) simulation. According to the experimental characteristics, the numerical simulation results reveal that the interface trap states, bulk tail states, and deep-level sub-gap defect states originating from oxygen-vacancy- (V_o_) related defects can be suppressed by an appropriate amount of N dopant. Correspondingly, the electrical properties and reliability of the a-IGZO TFTs are dramatically enhanced. In contrast, it is observed that the interfacial and deep-level sub-gap defects are increased when the a-IGZO TFT is doped with excess nitrogen, which results in the degeneration of the device’s performance and reliability. Moreover, it is found that tail-distributed acceptor-like N-related defects have been induced by excess N-doping, which is supported by the additional subthreshold slope degradation in the a-IGZO TFT.

## 1. Introduction

Currently, the backplane technology of amorphous InGaZnO (a-IGZO) thin-film transistors (TFTs) is attracting great attention for its use in pixel switching and driving units for next-generation display applications. The competitive advantage of a-IGZO TFT technology is that it can offer high current-driving capacity, high optical transparency, low power consumption and low process temperature compared with traditional Si-based TFTs [1,2,3]. Although a-IGZO TFT technology has made remarkable progress since it was first proposed by Nomura et al. in 2004, these devices still cannot achieve the desired performance and reliability due to high-density sub-gap states existing in the bandgap of a-IGZO [4,5]. It has been demonstrated that the sub-gap defects mainly originate from oxygen-vacancy-related (V_o_-related) defects induced by the structural disorder in a-IGZO [5,6,7], which degrades the electrical properties and reliability of TFTs by trapping electrons or holes in the channel layer and interfacial region under bias, light, and thermal stress [8,9,10,11]. To enhance the device performance and reliability, an in-situ nitrogen-doping (N-doping) approach during the a-IGZO active layer deposition has been proposed to suppress V_o_ defect generation [12,13,14]. For example, it has been demonstrated that N-doping can significantly improve the reliability of a-IGZO TFTs under positive gate-bias stress (PBS) and PBS with light illumination, since the N incorporated into a-IGZO will occupy the V_o_ sites and suppress V_o_-related defect generation [14,15]. Moreover, it has also been reported that the device performance and reliability are simultaneously enhanced by N and H co-doping, which is ascribed to the passivation of the V_o_ distributed at the active layer and interface region by forming N-–H and Zn–N bonds [16]. Although V_o_-related defects can be efficiently passivated by N-doping, a fundamental physical understanding of the impact of N-doping on the distribution of sub-gap states in a-IGZO TFTs is lacking. Since the device performance and reliability basically depend upon the nature and density of sub-gap defect states [4,17], an in-depth systematic study of the impact of N-doping on the sub-gap density of states (DOS) in a-IGZO TFTs is the key to future process improvement and optimization.

In this work, the influence of N-doping on the sub-gap V_o_-related defects in a-IGZO TFTs is qualitatively analyzed using technology computer-aided design (TCAD) simulation [18]. It is found that the density of the interface V_o_ trap states, bulk V_o_-related tail states and deep-level V_o_-related defect states of a-IGZO TFTs are significantly decreased by moderate N-doping, which is validated by the improvement in electrical properties and stability during PBS and sub-band illumination. In contrast, the DOS of a-IGZO TFT is increased when the a-IGZO TFTs are doped with excessive nitrogen atoms, which causes degeneration of the device performance and reliability. Meanwhile, it is confirmed that the tail-distributed acceptor-like N-related defects are formed by excessive N-doping, which leads to the degradation of subthreshold slope (*SS*) in a-IGZO TFT.

## 2. Experiments and Modeling Scheme

**Figure 1:**
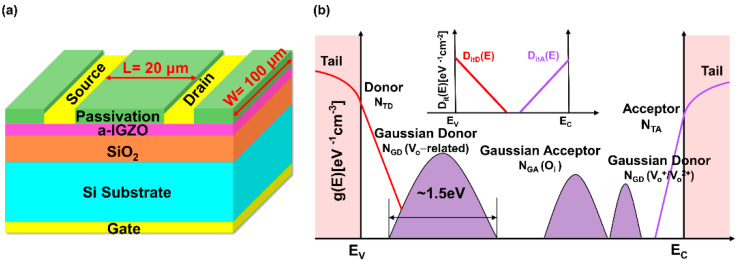
(**a**) Schematic diagram of the a-IGZO TFT with a bottom-gate structure; (**b**) Schematic illustration of the DOS model in the a-IGZO TFTs. The *N_TD_* and *N_TA_* represent the donor-like and acceptor-like tail states, respectively. The three gaussian curves represent the deep V_o_-related states (*N_GD_*(*V_o_-related*)**), oxygen interstitials (*N_GA_*(*O_i_*)**), and shallow donor states (*N_GD_*(*V_o_^+^/V_o_*^2+^)), respectively. The inset is the schematic illustration of the interface trap density (*D_it_*(*E*)).

The a-IGZO TFT structure used for numerical simulation is shown in Figure 1a. The devices in this work were fabricated on n-type Si substrate. First, the gate insulator was composed of a 200 nm SiO_2_ thin film grown by plasma-enhanced chemical vapor deposition (PECVD) with a rate of ~50 nm/min at 350 °C. The 45-nm-thick a-IGZO thin films were then deposited by direct-current (DC) sputtering system with a various gas mixture of N_2_/(O_2_ + N_2_) = 0%, 20%, and 40% at a fixed Ar flow rate of 30 sccm. The composition of the ceramic target used was In:Ga:Zn = 2:2:1 in atomic ratio. Subsequently, the device active region was patterned by conventional photolithography and wet chemical etching. Next, the Ti/Au (30/70 nm) bi-layer drain/source contact electrodes were evaporated by e-beam evaporation, and is the active region was further patterned using lift-off technique, which resulted in the final device dimensions of W/L = 100 μm/20 μm. Finally, a 100-nm-thick SiO_2_ passivation layer was deposited by PECVD. The fabricated devices were annealed in ambient air for 1 h at 300 °C.

In the Silvaco TCAD Simulation tool, ATLAS, a physics-based device simulator, is used to perform the electrical characterization, which can reduce the cost and time needed for experimentation [19]. It is also a powerful tool to predict the electrical behavior of specified semiconductor structures by using the Poisson and the continuity equations, which describe the electronic phenomena and electrical transport mechanism [19]. Based on the DOSs model of the a-IGZO TFTs, the types of the sub-gap states in the TFT channel region and interface region are illustrated in Figure 1b. In the a-IGZO material, the sub-gap states are mainly classified as acceptor-like and donor-like states, which can be depicted by Gaussian distribution states and exponentially decaying band-tail states. The specific mathematical model is expressed as follows [20,21,22]:

(1) $$g_{TA}\left(E\right)=N_{TA}\exp\left(\frac{E-E_{C}}{W_{TA}}\right)$$

(2) $$g_{TD}\left(E\right)=N_{TD}\exp\left(\frac{E_{V}-E}{W_{TD}}\right)$$

(3) $$g_{GA}\left(E\right)=N_{GA}\exp\left[-{\left(\frac{E_{GA}-E}{W_{GA}}\right)}^{2}\right]$$

(4) $$g_{GD}\left(E\right)=N_{GD}\exp\left[-{\left(\frac{E-E_{GD}}{W_{GD}}\right)}^{2}\right]$$

where the $g_{TD}\left(E\right)$ and $g_{TA}\left(E\right)$ denote the density of donor-like tail and acceptor-like tail states. The $g_{GA}\left(E\right)$ and $g_{GD}\left(E\right)$ represent the Gaussian-distributed acceptor-like and donor-like states. The $N_{TA}$ and $N_{TD}$ are the effective density at the conduction band minimum (*E_C_*) and valence band maximum (*E_V_*), respectively. The $W_{TD}$ and $W_{TA}$ are the characteristic slope energy of valence-band tail states and conduction-band tail states. The $N_{GD}$ and $N_{GA}$ are the total density of Gaussian donor and acceptor states, respectively. The $E_{GA}$ and $E_{GD}$ are the corresponding peak energy. $W_{GA}$ and $W_{GD}$ are the corresponding characteristic decay energy.

In addition, the interface trap density ($D_{it}\left(E\right)$) at the a-IGZO/dielectric interfacial region can be described as [23]:

(5) $$D_{it}\left(E\right)=D_{itA}\exp\left(\frac{E-E_{C}}{W_{itA}}\right)+D_{itD}\exp\left(\frac{E_{V}-E}{W_{itD}}\right)$$

where $D_{itD}$ and $D_{itA}$ represent the donor-like and acceptor-like interface trap density, respectively. The $W_{itA}$ and $W_{itD}$ denote the corresponding slope energy.

## 3. Results and Discussion

**Figure 2:**
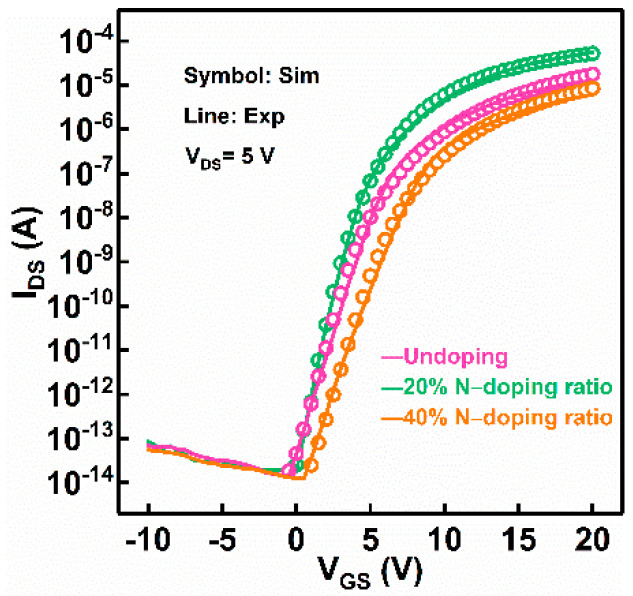
Simulated transfer characteristics for a-IGZO TFTs with different N-doping conditions: undoped, 20% N-doping ratio, and 40% N-doping ratio.

Figure 2 shows the simulated and experimental transfer characteristics of the a-IGZO TFTs under various N-doping conditions at V_DS_ = 5 V. The simulation results, in consistency with the experimental data, were achieved by calibrating the *D_itA_*, *N_TA_*, *N_GA_*(*O_i_*) and *N_GD_*(*V_o_^+^/V_o_*^2+^), and the simulation parameters are extracted and summarized in Table 1. The total trap density of the 20% N-doping ratio a-IGZO TFT was significantly decreased compared to the undoped a-IGZO TFT. For example, the *D_itA_* is decreased from 2.5 × 10^13^ eV^−1^ cm^−2^ to 8.0 × 10^12^ eV^−1^ cm^−2^, and the *N_TA_* was reduced from 8.0 × 10^19^ eV^−1^ cm^−3^ to 1.0 × 10^19^ eV^−1^ cm^−3^. Correspondingly, the subthreshold slope (*SS*) was decreased from 0.8 V/dec to 0.6 V/dec, and the threshold voltage (V_th_) was reduced from 5.0 V to 3.8 V. It has been demonstrated that the interface states and bulk traps in a-IGZO TFTs mainly originate from V_o_-related defects [5,15,24]. Therefore, the simulation results confirm that the improved electrical properties of a-IGZO TFTs can be ascribed to the suppression of the generation of V_o_-related defects in the device channel and interface region by N-doping. In contrast, when the N-doping ratio was increased to 40%, the number of total trap states was increased compared to the 20% N-doping ratio TFT, as shown in Table 1. Meanwhile, it was observed that the *SS* and V_th_ of the 40% N-doping ratio device were increased to 0.9 V/dec and 7 V, respectively, which indicates that V_o_-related defects generate when the device is subjected to excessive N-doping. This result can be explained by the fact that the formation of N–Ga bonds is facilitated by heavy N-doping, which then suppresses the bonding of Ga–O in the a-IGZO thin films [14,25].

**Figure 3:**
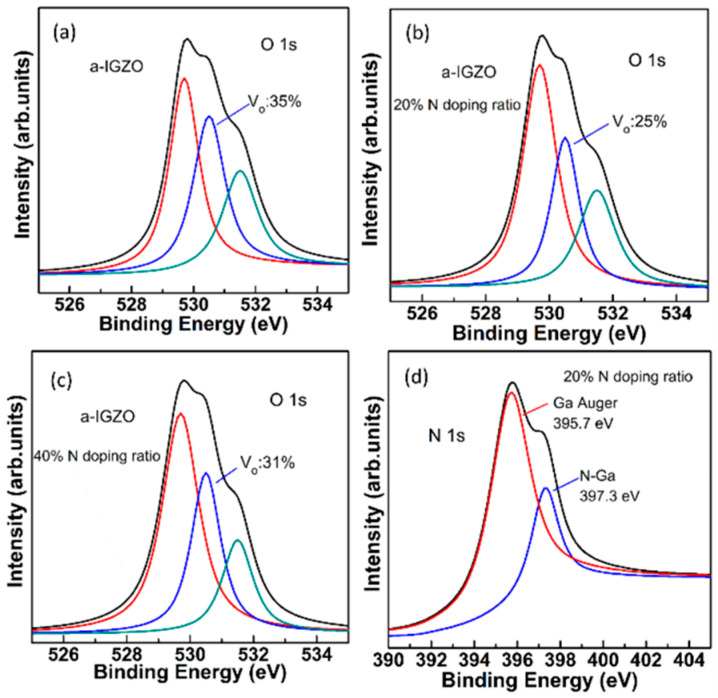
O 1s XPS spectra of the annealed a-IGZO films grown using N-doping ratio of (**a**) undoped, (**b**) 20% and (**c**) 40%. (**d**) N 1s XPS spectra of a-IGZO film grown with 20% N-doping ratio.

**Figure 4:**
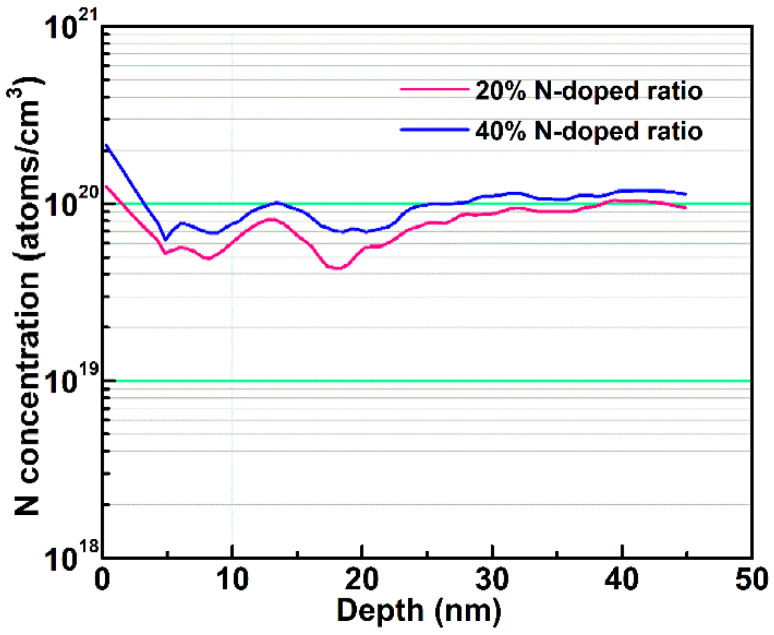
Depth profile of nitrogen in a-IGZO film deposited under 20% N-doping ratio and 40% N-doping ratio.

In addition, based on the simulation results, it was found that although the sub-gap DOS (*N_TA_*, *N_TD_*, *N_GD_*(*V_o_-related*)**, *N_GA_*(*O_i_*)**, and *N_GD_*(*V_o_^+^/V_o_*^2+^)) existing in the 40% N-doping ratio TFT was significantly higher than that of the 20% N-doping ratio TFT, the sub-gap DOS other than *N_TA_* was lower than that of the undoped TFT. Because the sub-band-gap density in a-IGZO film mainly originates from V_o_-related defects, the amount of V_o_ in annealed a-IGZO thin films with various N-doping ratios was analyzed by X-ray photoelectron spectroscopy (XPS). Figure 3a–c show the O 1 s XPS spectra of the a-IGZO films grown using different N-doped ratios. The binding energies were calibrated by taking the C 1s as reference at 284.6 eV. Gaussian fitting was applied to decompose the combined O 1s peak. The sub-peaks centered at binding energies of 529.7 eV, 530.5 eV, and 531.5 eV were attributed to O^2−^ ions surrounded by metal atoms (In, Ga and Zn), oxygen vacancies (V_o_), and OH^−^ impurities, respectively [26,27,28]. The relative level of V_o_ in a-IGZO film can be estimated by the proportion of peak area V_o_ to the whole O 1s (O_whole_). It was found that the area proportion of V_o_/O_whole_ was decreased from 35% for N-free a-IGZO film to 25% for a-IGZO film with the 20% N-doped ratio, as shown in Figure 3a,b, indicating that V_o_ decreases when the N is incorporated into the a-IGZO film. However, as shown in Figure 3c, it was found that the V_o_ increased to 31% for the a-IGZO film deposited with the 40% N-doped ratio, which means that the additional V_o_ was created when excess N atoms were doped into the a-IGZO film. Furthermore, the N 1s spectra XPS of the annealed a-IGZO film with 20% N-doped ratio was also analyzed, as shown in Figure 3d. The N 1 s spectrum is decomposed into two peaks at 395.7 and 397.3 eV, which are associated with the Ga Auger and N-Ga bonding [29], respectively. Therefore, the XPS results reveal that moderate N doping in a-IGZO film can suppress the generation of V_o_, and excess N incorporation into a-IGZO film leads to an increase in V_o_. Because the V_o_ existing in the 40% N-doping ratio a-IGZO film was lower than that of undoped a-IGZO film, the increased *N_TA_* in 40% N-doping ratio a-IGZO TFT should be the result of the generation of N-related defects by excess N-doping [16,30], which agrees well with the increased *SS* from 0.8 V/dec to 0.9 V/dec compared to undoped a-IGZO TFT. Meanwhile, to quantitatively estimate the N concentration in the a-IGZO active layer, the actual level of N-doping in annealed a-IGZO films is characterized by secondary ion mass spectrometry (SIMS) measurement [31,32,33]. Figure 4 shows the depth profile of N concentration in the a-IGZO film deposited with the 20% and 40% N-doped ratios. N is clearly detectable, and there is a considerable amount of incorporated nitrogen (~10^20^ cm^−3^) in the a-IGZO film. It has been reported that the value of the sub-gap density of states (DOSs) near the VBMs is about 5–9 × 10 ^20^ cm^−3^ in a-IGZO film [5,34]. In this work, it is found that when the concentration of N doping in the channel region of a-IGZO TFT with a 20% N doping ratio is ~1.0 × 10^20^ cm^−3^, the electrical performance and stability of the device are dramatically improved. But when the concentration of N-doping in the channel region of a-IGZO TFT with a 40% N doping ratio is increased to ~1.2 × 10^20^ cm^−3^, the electrical performance and stability of a-IGZO TFT are degraded.

**Figure 5:**
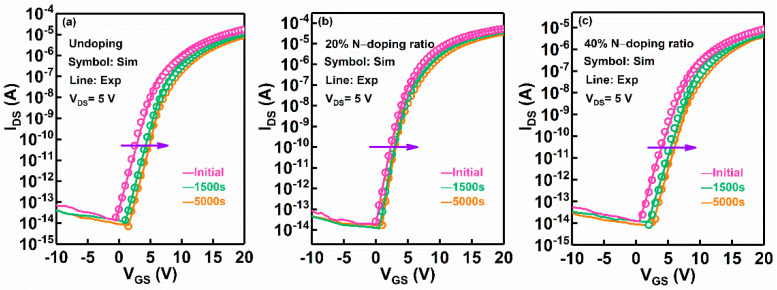
Simulated transfer characteristics against positive bias stress (PBS) time for the a-IGZO TFTs fabricated with different N-doping ratios: (**a**) undoped, (**b**) 20% N-doping ratio, and (**c**) 40% N-doping ratio.

According to the distribution of the sub-gap DOS fitted in a-IGZO TFTs with various N-doping ratios, a comprehensive quantitative study on the device stability under positive bias stress (PBS) was carried out. During the PBS process, the TFTs were applied at a V_GS_ of 15 V for the stress duration of 5000 s. Figure 5a–c show the experimental and simulated evolution of transfer characteristics as a function of PBS time for the a-IGZO TFTs with different N-doping ratios. It was found that the shift in threshold voltage (∆V_th_) induced by PBS was 2.06 V, 0.8 V, and 1.68 V for undoped a-IGZO TFT, 20% N-doping a-IGZO TFT, and 40% N-doping a-IGZO TFT, respectively. It has been reported that the shift in V_th_ (∆V_th_) of a-IGZO TFTs under PBS basically originates from the interfacial V_o_-related defects trapping electrons at the device interfacial region [35,36]. In the simulation results, it was clearly seen that the *D_it_*(*E*) for the 20% N-doping ratio a-IGZO TFT was lower than of the undoped a-IGZO TFT and 40% N-doping ratio a-IGZO TFT. For example, the *D_itA_* for undoped a-IGZO TFT, 20% N-doped a-IGZO TFT, and 40% N-doped a-IGZO TFT is 2.5 × 10^13^ eV^−1^ cm^−2^, 8.0 × 10^12^ eV^−1^ cm^−2^, and 1.5 × 10^13^ eV^−1^ cm^−2^, respectively, suggesting that interfacial V_o_-related defects can be suppressed by moderate N-doping.

**Figure 6:**
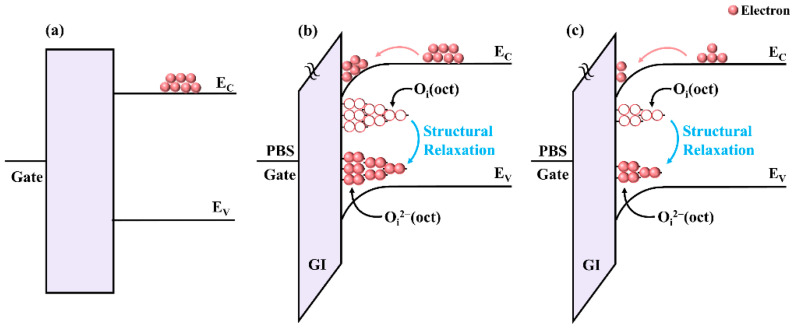
Schematic of the energy-band diagram of the a-IGZO TFTs. (**a**) the energy-band diagram of the TFTs before PBS, (**b**,**c**) the energy-band diagrams of the undoped TFTs and 20% N-doping ratio TFTs during PBS, respectively.

In addition, it has been reported that weak oxygen ions originating from structural disorder in a-IGZO TFTs cause ∆V_th_ under PBS [37]. During the PBS process, the weak oxygen ions are ionized to generate oxygen interstitials (O_i_) because of their low formation energies [4,38,39]. Meanwhile, according to the first-principle studies, the generated O_i_ during PBS forms an octahedral configuration [O_i_(oct)] and is electrically active. Correspondingly, the introduced O_i_(oct)-related defect states are distributed above the mid-gap (E_i_) in the a-IGZO TFTs [40]. When the Fermi level moves up under PBS, the O_i_(oct)-related states are filled by trapping electrons and thus negatively charged to generate O_i_^2−^oct) [41]. Figure 6a–c show the forming process of O_i_^2−^(oct)-related charged states in a-IGZO TFTs undergoing PBS. Because of the structural relaxation effect, the O_i_^2−^(oct)-related charged states are transformed into deep-level negative-U states, which are located below the mid-gap in the a-IGZO TFTs [37,42]. As a result, although new O_i_^2−^(oct)-related charged states are generated under the PBS process, the *SS* of a-IGZO TFTs has no apparent change due to the negative-U property of the O_i_^2−^(oct) states.

According to the simulation results, *N_GA_*(*O_i_*) exhibited an increasing trend for a-IGZO TFTs with various N-doping ratios under the PBS process, as shown in Table 2. For example, the *N_GA_*(*O_i_*) in the N-free a-IGZO TFT continuously increased from 2.6 × 10^17^ eV^−1^ cm^−3^ to 3.6 × 10^17^ eV^−1^ cm^−3^ after 5000 s PBS. This result shows that the O_i_(oct)-related defects were generated in the a-IGZO TFT upon PBS, originating from weak oxygen ions in the device channel region. Compared to the undoped a-IGZO TFT, the generated O_i_(oct)-related trap states under PBS in the 20% N-doping ratio a-IGZO TFT decreased from 3.6 × 10^17^ eV^−1^ cm^−3^ to 2.5 × 10^17^ eV^−1^ cm^−3^ after 5000 s PBS, due to the suppression of V_o_-related traps by N-doping. Correspondingly, the device had superior electrical reliability under PBS. In contrast, the generated O_i_(oct)-related trap states under PBS in the 40% N-doping ratio a-IGZO TFT (3.2 × 10^17^ eV^−1^ cm^−3^) were higher than that of the 20% N-doping ratio a-IGZO TFT, due to the V_o_-related traps generated by heavy N-doping, resulting in device-stability degeneration under PBS.

**Figure 7:**
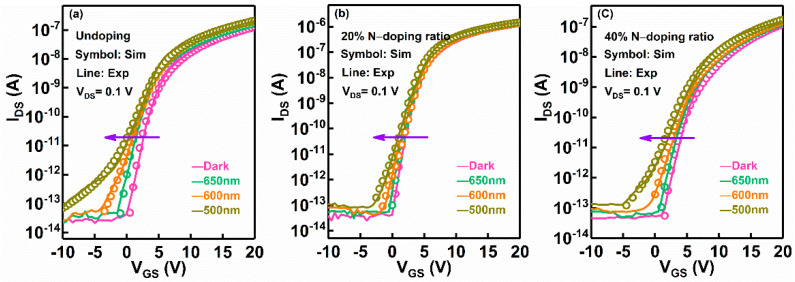
Simulated transfer characteristics against various monochromatic light illumination for the a-IGZO TFTs fabricated with different N-doping ratios: (**a**) undoping, (**b**) 20% N-doping ratio, and (**c**) 40% N-doping ratio.

**Figure 8:**
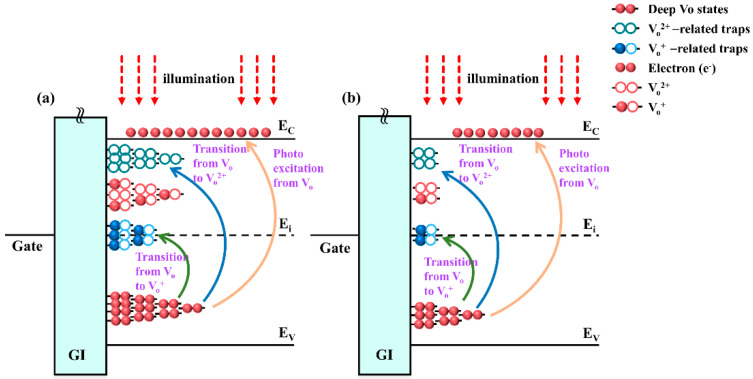
Schematic of the generation process of V_o_-related defect states under short-wavelength light illumination: (**a**) undoping, (**b**) 20% N-doping ratio.

Finally, to reveal the impact of N-doping on the distributions of deep-level sub-gap states, the transfer characteristics of a-IGZO TFTs with various N-doping conditions under sub-band-gap light illumination were simulated. The simulation results are shown in Figure 7a–c, and the simulation parameters are extracted in Table 3. It was found that the I-V curves of the undoped a-IGZO TFT exhibited an overall shift in a negative direction with the decrease in incident light wavelength from 650 nm to 500 nm, as shown in Figure 7a. The negative ΔV_th_ is attributable to the photorelease of occupied electrons in the interface states and deep-level sub-gap states. It has been demonstrated that the deep-level defects in a-IGZO mainly originate from neutral V_o_, which would be entirely occupied above E_V_ with an energy width of ~1.5 eV [5,24,43]. As a result, the occupied interfacial and deep-level V_o_-related defects would be ionized into *V_o_^+^/V_o_*^2+^ under the corresponding photon energy illumination, which agrees well with the simulation result that the *N_GD_*(*V_o_^+^/V_o_*^2+^) exhibits continuously increase as the illumination wavelength decreases [44,45], as shown in Table 3. It is clear that the *N_GD_*(*V_o_^+^/V_o_*^2+^) of the undoping a-IGZO TFT is increased from 1.5 × 10^17^ eV^−1^ cm^−3^ to 3.0 × 10^17^ eV^−1^ cm^−3^ with the decrease of incident light wavelength from 650 nm to 500 nm. In addition, based on the first-principle calculation and experimental observation, the activation energy (E_a_) of the photoexcited ionization process from occupied deep-level V_o_ to V_o_^+^ and V_o_^2+^ is required to be ~2.0 eV and ~2.3 eV, respectively [37,45]. Meanwhile, these photo-induced transitions (both V_o_ to V_o_^+^ and V_o_ to V_o_^2+^) could cause the outward relaxation in the vicinity of metal atoms, which lead to the generation of new defect level near the E_i_ and E_c_ edge [24,45]. The formation process of V_o_^+^ and V_o_^2+^ states induced by sub-band-gap illumination is illustrated in Figure 8. In the simulation, it is observed that the generated *N_GA_*(*V_o_^+^-related*)** near the mid-gap is 9.0 × 10^16^ eV^−1^ cm^−3^ at λ = 600 nm (~2.0 eV), and the generated *N_GD_*(*V_o_*^2+^*-related*)** near bottom of the conduction band is 1.2 × 10^17^ eV^−1^ cm^−3^ at λ = 500 nm (~2.3 eV). It has been reported that the variation of *SS* (∆*SS*) is in connection with the amount of created trap states (∆*N*_*t*_) in the TFTs channel and interface region, which is expressed by using the following equation [45]:

(6) $$\Delta SS=\frac{\Delta N_{t}\ln\left(10\right)kT}{C_{i}}$$

where *k* is the Boltzmann’s constant, *T* is the absolute temperature, *C_i_* is the capacitance of the gate dielectric. Therefore, the *SS* degradation for the undoping a-IGZO TFT induced by incident illumination of λ ≤ 600 nm is caused by the new defects creation near the E_i_ and E_c_ edge.

Comparatively, it is found that the density of deep-level V_o_-related traps is suppressed by moderate N-doping into the a-IGZO TFT. As shown in Table 3, the *N_GD_*(*V_o_-related*) in the 20% N-doping ratio a-IGZO TFT is decreased from 8.0 × 10^20^ eV^−1^ cm^−3^ to 5.0 × 10^20^ eV^−1^ cm^−3^ compared with the undoping a-IGZO TFT, and the generated *N_GD_*(*V_o_*^2+^*-related*) is reduced from 1.2 × 10^17^ eV^−1^ cm^−3^ to 7.0 × 10^16^ eV^−1^ cm^−3^ under λ= 500 nm. Correspondingly, the electrical stability of the 20% N-doping ratio a-IGZO TFT under light illumination is significantly improved. It is found that the ∆*SS* and ∆V_th_ in the 20% N-doping ratio a-IGZO TFTs (0.58 V/dec; −0.8 V) are lower than that of undoping a-IGZO TFT (1.95 V/dec; −1.6 V) at λ = 500 nm, as shown in Figure 7b. Therefore, it can be concluded that the improved device reliability under sub-band light illumination is owing to the passivation of V_o_-related traps in the device channel region by moderate N-doping. However, the deep-level V_o_-related defect in the 40% N-doping ratio a-IGZO TFT is increased to 6.5 × 10^20^ eV^−1^ cm^−3^ compared with the 20% N-doping ratio a-IGZO TFT, and the generated *N_GD_*(*V_o_*^2+^*-related*) is increased to 9.0 × 10^16^ eV^−1^ cm^−3^ under λ = 500 nm. Meanwhile, the degradation of *SS* and V_th_ (1.5 V/dec; −1.3 V) are observed in the 40% N-doping ratio a-IGZO TFT. This result means that superfluous N-doping into the a-IGZO TFTs will result in the increase of deep-level V_o_ traps [25], which degrades the device stability under sub-band-gap illumination.

## 4. Conclusions

In this work, the fundamental physical understanding of the N-doping on DOS over the whole sub-band-gap range has been analyzed by Silvaco TCAD simulation. It is found that the improved electrical performances for the 20% N-doping ratio a-IGZO TFT are owing to the suppression of interface V_o_ trap states and bulk tail states (V_o_-related) by N-doping. Meanwhile, the O_i_ and deep-level V_o_-related traps are suppressed by an appropriate amount of N dopant, which causes the improvement of device stability during PBS and sub-band illumination processes by suppressing the formation of O_i_ and the photoexcited ionization from occupied deep-level V_o_ to V_o_^+^ and V_o_^2+^, respectively. In contrast, the excessive N-doping will cause the generation of acceptor-like N-related defects and the increase of V_o_-related traps in the channel and interface region of a-IGZO TFTs, which leads to the degeneration of the device performance and reliability.

## Figures and Tables

**Table 1:** Densities of key defect model parameters for a-IGZO TFT fitted after different N-doping ratios.

Parameters	Undoping	20% N-Doping Ratio	40% N-Doping Ratio	Description
D_itA_ (eV^−1^ cm^−2^)	2.5 × 10^13^	8.0 × 10^12^	1.5 × 10^13^	Acceptor-like interface trap densities
D_itD_ (eV^−1^ cm^−2^)	3.0 × 10^13^	9.0 × 10^12^	2.0 × 10^13^	Donor-like interface trap densities
N_TA_ (eV^−1^ cm^−3^)	8.0 × 10^19^	1.0 × 10^19^	1.5 × 10^20^	Acceptor-like tail states at E = Ec
N_TD_ (eV^−1^ cm^−3^)	1.5 × 10^20^	8.0 × 10^19^	1.3 × 10^20^	Donor-like tail states at E = Ev
N_GD_(V_o_-related) (eV^−1^ cm^−3^)	8.0 × 10^20^	5.0 × 10^20^	6.5 × 10^20^	Peak of V_o_-related states
N_GA_(O_i_) (eV^−1^ cm^−3^)	2.6 × 10^17^	1.4 × 10^17^	2.1 × 10^17^	Peak of O_i_ states
N_GD_(V_o_^+^/V_o_^2+^) (eV^−1^ cm^−3^)	8.0 × 10^16^	5.0 × 10^16^	6.5 × 10^16^	Peak of V_o_^+^/V_o_^2+^ states

**Table 2:** Densities of key defect model parameters for a-IGZO TFT fitted with different N-doping ratios after PBS.

Parameters	N-Doping Ratio	Initial	1500 s	5000 s	Description
N_GA_(O_i_) (eV^−^^1^ cm^−^^3^)	0%	2.6 × 10^17^	3.2 × 10^17^	3.6 × 10^17^	Peak of O_i_ states
	20%	1.4 × 10^17^	2.0 × 10^17^	2.5 × 10^17^	
	40%	2.1 × 10^17^	2.8 × 10^17^	3.2 × 10^17^	

**Table 3:** Densities of key defect model parameters for a-IGZO TFT fitted with different N-doping ratios after monochromatic light illumination.

Parameters	N-Doping Ratio	Dark	650 nm	600 nm	500 nm	Description
N_GD_(V_o_-related) (eV^−1^ cm^−3^)	0%	8.0 × 10^20^				Peak of V_o_-related states
	20%	5.0 × 10^20^				
	40%	6.5 × 10^20^				
N_GD_(V_o_^+^/V_o_^2+^) (eV^−1^ cm^−3^)	0%	8.0 × 10^16^	1.5 × 10^17^	2.5 × 10^17^	3.0 × 10^17^	Peak of V_o_^+^/V_o_^2+^ states
	20%	5.0 × 10^16^	9.0 × 10^16^	1.5 × 10^17^	2.2 × 10^17^	
	40%	6.5 × 10^16^	1.2 × 10^17^	2.0 × 10^17^	2.5 × 10^17^	
N_GD_(V_o_^2+^-related) (eV^−1^ cm^−3^)	0%	—	—	—	1.2 × 10^17^	Peak of V_o_^2+^-related states
	20%	—	—	—	7.0 × 10^16^	
	40%	—	—	—	9.0 × 10^16^	
N_GA_(V_o_^+^-related) (eV^−1^ cm^−3^)	0%	—	—	9.0 × 10^16^	9.0 × 10^16^	Peak of V_o_^+^-related states
	20%	—	—	4.0 × 10^16^	4.0 × 10^16^	
	40%	—	—	7.5 × 10^16^	7.5 × 10^16^	
